# Dietary fat composition influences glomerular and proximal convoluted tubule cell structure and autophagic processes in kidneys from calorie‐restricted mice

**DOI:** 10.1111/acel.12451

**Published:** 2016-02-08

**Authors:** Miguel Calvo‐Rubio, Mª Isabel Burón, Guillermo López‐Lluch, Plácido Navas, Rafael de Cabo, Jon J. Ramsey, José M. Villalba, José A. González‐Reyes

**Affiliations:** ^1^ Departamento de Biología Celular, Fisiología e InmunologíaCampus de Excelencia Internacional AgroalimentarioceiA3Universidad de CórdobaCórdobaSpain; ^2^ Centro Andaluz de Biología del DesarrolloCIBERERInstituto de Salud Carlos IIIUniversidad Pablo de Olavide‐CSICSevillaSpain; ^3^ Translational Gerontology BranchNational Institute of AgingNational Institutes of HealthBaltimoreMDUSA; ^4^ VM Molecular BiosciencesUniversity of CaliforniaDavisCAUSA

**Keywords:** aging, calorie restriction, dietary fat, kidney, mice

## Abstract

Calorie restriction (CR) has been repeatedly shown to prevent cancer, diabetes, hypertension, and other age‐related diseases in a wide range of animals, including non‐human primates and humans. In rodents, CR also increases lifespan and is a powerful tool for studying the aging process. Recently, it has been reported in mice that dietary fat plays an important role in determining lifespan extension with 40% CR. In these conditions, animals fed lard as dietary fat showed an increased longevity compared with mice fed soybean or fish oils. In this paper, we study the effect of these dietary fats on structural and physiological parameters of kidney from mice maintained on 40% CR for 6 and 18 months. Analyses were performed using quantitative electron microcopy techniques and protein expression in Western blots. CR mitigated most of the analyzed age‐related parameters in kidney, such as glomerular basement membrane thickness, mitochondrial mass in convoluted proximal tubules and autophagic markers in renal homogenates. The lard group showed improved preservation of several renal structures with aging when compared to the other CR diet groups. These results indicate that dietary fat modulates renal structure and function in CR mice and plays an essential role in the determination of health span in rodents.

## Introduction

Aging can be defined as a time‐dependent degenerative process caused by accumulated damage that leads to cell dysfunction, tissue failure, and death (Campisi, 2013). Although the action of free radicals (many of them produced at the mitochondria) may contribute to aging, the mechanisms through which this occurs are still not entirely known. Recently, several hallmarks have been proposed to explain the molecular and physiological basis of aging and mitochondrial dysfunction seems to play a central role in this process (López‐Otín *et al*., 2013; González‐Freire *et al*., 2015).

To analyze the physiological basis of aging, several experimental procedures have been developed. Among them, the reduction of calorie intake without malnutrition, also known as calorie restriction (CR), has been shown to be the most robust nongenetic or pharmacological approach to study this phenomenon (Weindruch & Walford, 1988). A reduction in calorie intake (typically 20–40% of the *ad libitum* fed controls) has been reported to increase lifespan and to prevent cancer, diabetes, hypertension, and other age‐related diseases in a wide range of animals, including non‐human primates and humans (Colman *et al*., 2009; Mattison *et al*., 2012). Although the mechanisms by which CR operates are not completely understood, it is often assumed that the anti‐aging action of CR is partially based on its ability to suppress oxidative stress and maintain the cellular redox status to provide optimal cell signaling processes and normal gene expression (Chung *et al*., 2013). Also, CR has been proposed to induce biogenesis of efficient mitochondria (López‐Lluch *et al*., 2008).

We have recently confirmed that the composition of dietary fat modulates longevity of mice fed CR diets (López‐Domínguez *et al*., 2015a). Animals fed a 40% CR diet with lard (CRL, high in saturated and monounsaturated fatty acids) as the primary dietary fat had extended lifespan compared to CR animals consuming diets with either soybean oil (CRS, high in n‐6 polyunsaturated fatty acids, PUFAs) or fish oil (CRF, high in n‐3 PUFAs) as the primary lipid sources (López‐Domínguez *et al*., 2015a). However, the influence of dietary fat composition on physiological function and health span are not known.

According to the ‘Mitochondrial Free Radical Theory of Aging’ (Miquel *et al*., 1980), accumulation of reactive oxygen species (ROS) in mitochondria (the subcellular organelle with the highest rate of ROS production) results in damage not only to mitochondrial DNA and proteins, but also to membrane phospholipids, and this oxidative damage is decreased in animals maintained on CR (Youngman *et al*., 1992; Pamplona *et al*., 2002). An inverse correlation between lifespan and the degree of membrane phospholipid unsaturation has been proposed (Pamplona *et al*., 2002; Hulbert, 2003), with PUFAs being more susceptible to peroxidation and other modifications which result in the accumulation of oxidative injury in membranes containing these fatty acids. The decreased content of long‐chain PUFAs in mitochondria isolated from different organs after CR seems to support this idea (Yu *et al*., 2002). According to these results, we hypothesized that the extended longevity found in the CRL‐fed group correlates with the higher MUFA content in mitochondria from these animals as detected in hepatocytes and skeletal muscle (López‐Domínguez *et al*., 2013, 2015b). Furthermore, the decreased susceptibility of membranes to phospholipid peroxidation may improve mitochondrial function, a phenomenon likely linked to lifespan extension (Jové *et al*., 2014).

Kidney has been considered as an essential organ to understand the aging process, and renal function has been suggested to be one of the major predictors of longevity (Hediger, 2002). Thus, as renal function declines with age, several structural and functional changes have been reported to occur in renal glomeruli and in proximal convoluted tubule (PCT) epithelial cells (see, for example, Martin & Sheaff, 2007; Wiggins, 2012; Bolignano *et al*., 2014). However, relatively little is known about the influence of dietary fats on changes in renal structure and function in CR animals.

Besides the potential role of CR in mitigating oxidative stress, autophagy is also a crucial phenomenon to explain CR effects on longevity and healthy aging (Rajawat *et al*., 2009; Speakman & Mitchell, 2011 and Madeo *et al*., 2015). By this mechanism, different aged subcellular structures which accumulate molecular damage are degraded through a lysosomal pathway and the resulting products are released into the cytosol for recycling or to supply energy during starvation periods (Cuervo, 2004). Deregulation of autophagy has been shown to be involved in the pathogenesis of a number of renal disorders, many of them directly related to aging (Huber *et al*., 2012). Kidney aging markers were delayed or even reversed by CR (Wiggins *et al*., 2005; McKiernan *et al*., 2007), but little is known about the impact of the different dietary constituents on kidney aging in CR mice. In this study, we analyze structural and ultrastructural changes in kidney mediated by different dietary fats in CR mice. Moreover, in an attempt to establish possible links between healthy renal aging and health span expansion mediated by dietary fat under CR conditions, we have also analyzed changes in the expression of proteins related to mitochondrial biogenesis and autophagy processes.

## Results

### Physiological parameters and p16 expression levels

Body weight and urea and creatinine serum levels after 6 and 18 months of dietary intervention are shown in Table 1. As expected, mice sequentially lost weight in the CRS compared to the control (CON) group and there were no differences between CR groups at either 6 or 18 months (Table 1). A similar result was found for serum urea levels, which were decreased in the CRS group at both time periods. Under CR, dietary fat did not induce changes in this parameter. On the other hand, creatinine levels increased in CRL after 6 months of intervention but decreased to reach similar values to the other CR groups at 18 months. As occurred for urea, dietary fat did not induce significant changes among CR groups at 18 months (Table 1).

**Table 1:** Body weights and serum urea and creatinine levels in mice fed control (CON) or 40% calorie‐restricted diets (CRL, CRS and CRF) after 6 and 18 months of dietary intervention. Data are expressed as mean values ± SE

	CON		CRL		CRS		CRF	
	6 months	18 months	6 months	18 months	6 months	18 months	6 months	18 months
Weight (g)	37.70 ± 1.33^a^	32.56 ± 2.58	26.71 ± 0.78	28.19 ± 0.75	26.73 ± 1.1^b^	28.86 ± 1.53	28.02 ± 1.19	28.67 ± 0.94
Serum urea (mg dL^−1^)	39.63 ± 1.43^c^	36.24 ± 2.14^c^	31.73 ± 1.19	32.96 ± 3.87	31.8 ± 1.85	29.32 ± 1.82	28.73 ± 2.64	31.85 ± 1.77
Serum creatinine (mg dL^−1^)	0.68 ± 0.06	0.61 ± 0.12	0.85 ± 0.08^d^	0.54 ± 0.07	0.61 ± 0.07	0.61 ± 0.06	0.50 ± 0.09	0.63 ± 0.07

a *P* < 0.05 vs CON 18 months and *P* < 0.01 vs CRS 6 months.

b *P* < 0.05 vs CRS 18 months.

c *P* < 0.01 vs CRS 6 months.

d *P* < 0.01 vs CRL 18 months and *P* < 0.05 vs CRS and CRF 6 months.

**Figure 1:**
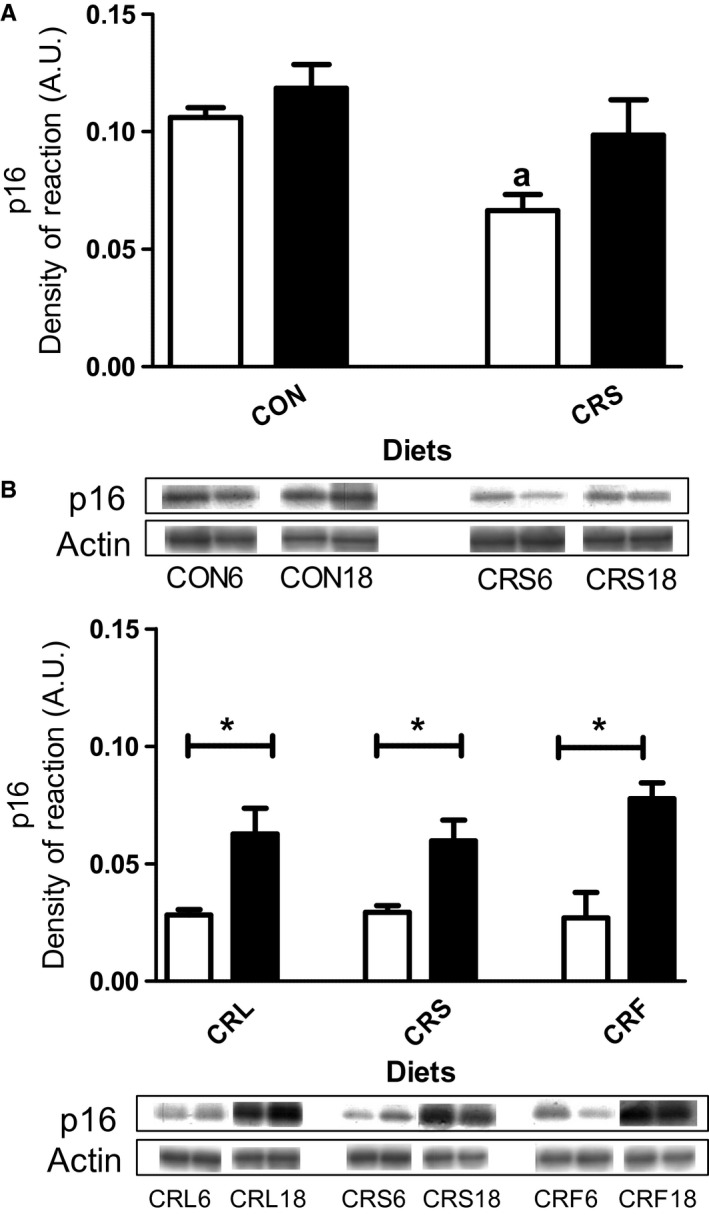
Protein expression levels of p16. Panel A shows CON and CRS mice (^a^ *P* < 0.05 vs CON after 6 months of dietary intervention) and panel B the three CR diet groups (*P < 0.5). In all figures, white bars refer to 6 and black bars to 18 months of dietary intervention. In panels A and B, two representative Western blot bands for each experimental group are shown.

The expression level of p16, a marker of tissue aging, changed during CR. Six months of CR induced a decrease in this parameter when compared with the CON group, but no differences were found between CRS and control (CON) mice at 18 months (Fig. 1A). When comparing the different CR groups, a significant increase in p16 expression level was found at 18 versus 6 months regardless of the dietary fat source (see Fig. 1B) but no changes were detected in 6 or 18 months when comparing among the different CR groups (Fig. 1B).

### Renal corpuscle and glomerular filtration barrier morphology

**Figure 2:**
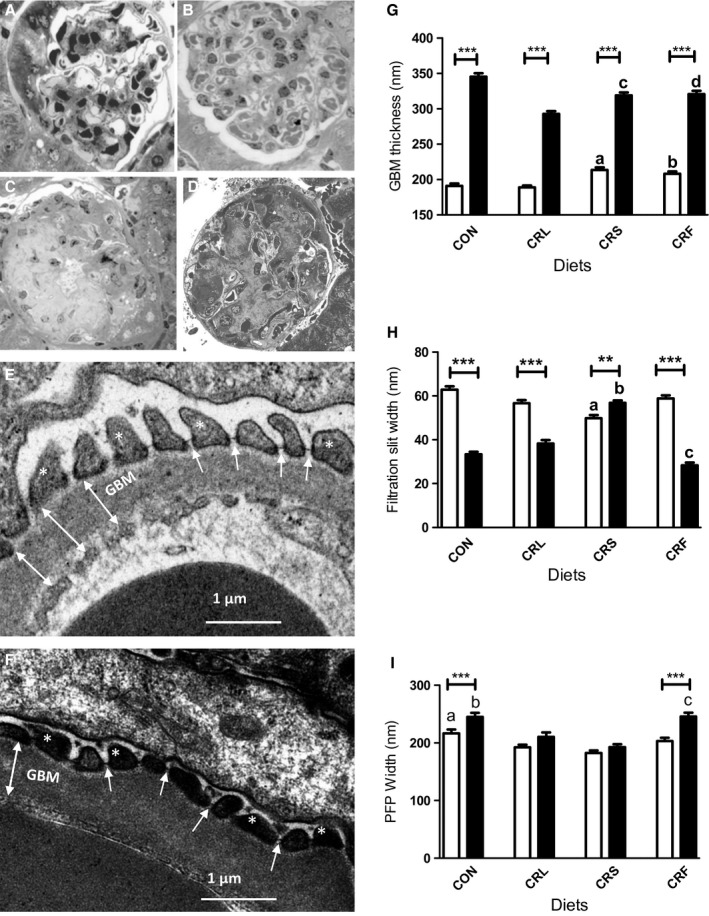
Analysis of structural and ultrastructural features of renal glomeruli in CON and CR mice. Panels A to D show light microscopy pictures of glomerular structure after 6 (A, CON; B, CRS) and 18 months of intervention (C, CON and D, CRS). Panels E and F show examples of ultrastructural modifications of glomerular basement membrane (GBM), filtration slits (FS) (white arrows), and podocyte foot processes (asterisks) width after 18 months of intervention (E, CRS and F, CRF). In panel E, we show three examples of how measurements of GBM thickness were taken (two‐headed arrows). The results of quantification are included in panels G (GBM), H (FS), and I (PFP). Aging induced striking changes in all of these structures (**P < 0.01 and ***P < 0.001) when comparing 6 vs 18 months in the same diet group. In panel G, ^a^ *P* < 0.001 vs CON and CRL; ^b^ *P* < 0.001 vs CRL after 6 months of intervention; ^c^ *P* < 0.001 vs CON and CRL and ^d^ *P* < 0.001 vs CRL after 18 months of intervention. In panel H, ^a^ *P* < 0.001 vs all the other 6‐month‐old groups; ^b^ *P* < 0.001 vs all other 18‐month‐old groups; ^c^ *P* < 0.001 vs CRL and CRS in 18‐month‐old animals. In panel I, ^a^ *P* < 0.001 vs CRS after 6 months of intervention; ^b^ *P* < 0.001 vs CRS after 18 months of CR, and ^c^ *P* < 0.001 vs CRS and CRL after 18 months of CR.

Examination of semi‐thick sections of renal cortex from 6‐month‐old CON mice allowed us to visualize renal corpuscles showing a typical morphology. When comparing corpuscle structure in CON group with that obtained from CRS mice after 6 months of CR, no changes were detected since virtually all the glomeruli showed an unaltered morphology (Fig. 2A,B). However, in 18‐month‐old CON and CRS animals, sections showed a high number of glomerulosclerosis figures (about 50–55%) with no appreciable differences in percentage among these two dietary groups (Fig. 2C,D). However, an ultrastructural analysis performed on glomeruli with unaltered morphology showed striking differences between CON and CRS mice.

Glomerular basement membrane (GBM) thickness was measured on high‐magnification electron micrographs (see Fig. 2E,F), and the results are depicted in Fig. 2G. Aging resulted in a significant increase (*P* < 0.001) of this parameter in all of the diet groups. Differences were also found when comparing control and CRS diets at 6 and 18 months of CR. After 6 months, dietary fat also induced changes in GBM thickness among the CR groups. Thus, a significant increase was found in CRS‐ and CRF‐fed animals in comparison with CRL, which showed the lowest value. When comparing animals at 18 months of CR, we found that basal membrane enlargement due to aging was reduced to different degrees depending on the dietary fat, with CRL mice showing the thinnest GBM in their glomeruli compared to the others CR groups (see Fig. 2G).

Glomerular filtration slits (FS), which can be observed as narrow spaces between podocyte processes (see Fig. 2E,F), were also measured, and the results are displayed in Fig. 2H. In CON mice, aging induced a significant decrease in this parameter that was not observed in CRS group where FS width increased with age (Fig. 2H). Similarly, all CR groups showed a decrease in FS width with aging. Of note, the decrease observed in the CRL groups was not as prominent as occurred in the CRF group. In 6‐month‐old mice, FS showed similar widths regardless of diet except for CRS in which mean slit width was significantly decreased compared to all the other dietary groups. However, after 18 months of CR we found the highest values for FS among CR groups in the CRS and the lowest in CRF group (Fig. 2H).

Finally, we measured podocyte foot processes (PFP) width in the zone of contact with the GBM (see Material and Methods and Fig. 2I). PFP width increased in CON animals during aging (Fig 2I). Six months of CR induced decreased PFP although this parameter was also increased with aging. Nevertheless, in aged animals fed under CR PFP widths, values were not as high as in CON mice. On the other hand, lard or fish oil as dietary fat had differential results compared to CRS group. Thus, in CRF this parameter significantly increased during aging but in CRL mean PFP width remained unaltered (Fig. 2I).

Using the data obtained for GBM, FS, and PFP, we performed statistical analyses to assess possible correlations between size alterations of these structures. Thus, a significant negative correlation between GBM thickness and FS width was found both in 6‐ and 18‐month interventions, and, interestingly, a similar result was obtained when comparing FS with PFP. However, PFP width and GBM thickness positively correlated in all of the dietary groups. These correlations seem to be independent of the dietary fat but clearly depend on the animal age (see Fig. S4A,B,C).

### Mitochondrial mass and ultrastructure in PCT cells

**Figure 3:**
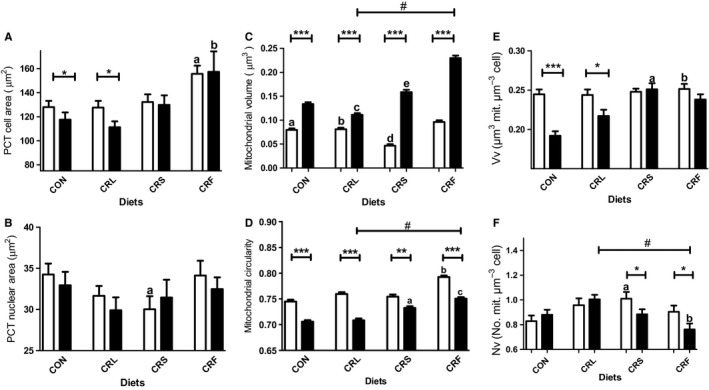
Ultrastructural features of PCT epithelial cells (A) and nuclei (B), and mitochondrial planimetric (C and D) and stereological analysis (E and F) in PCT cells from the different dietary groups. In all panels *P < 0.05; **P < 0.01, and ***P < 0.001. In panel A, ^a^ *P* < 0.01 and 0.05 vs CRL and CRS in 6‐month‐old mice, respectively, and ^b^ *P* < 0.001 and 0.05 vs CRL and CRS in 18‐month‐old mice, respectively. In panel B, ^a^ *P* < 0.05 vs CRF after 6 months of intervention. Mitochondrial volume (C) and circularity coefficient (D) changed depending on age and dietary fat in CR groups. In panel C, ^a^ *P* < 0.001 vs CRS 6 months; ^b^ *P* < 0.001 and *P* < 0.01 vs CRS and CRF, respectively, after 6 months of CR;^c^ *P* < 0.001 vs CRS and CRF 18 months; ^d^ *P* < 0.001 vs CRF six months and ^e^ *P* < 0.001 vs CRF 18‐month‐old group (# denotes a linear trend CRL < CRS < CRF in 18‐month‐old animals; *P* < 0.001). In panel D, ^a^ *P* < 0.001 vs CON and CRL;^b^ *P* < 0.001 vs CRS and ^c^ *P* < 0.001 vs CRS and CRL in 18‐month mice (# denotes a linear trend of increased mitochondrial circularity coefficient CRL < CRS < CRF). The stereological parameters Vv and Nv are represented in E and F, respectively. In E, ^a^ *P* < 0.01 vs CRL and CON in 18‐month‐old animals and ^b^ *P* < 0.01 vs CRL in 6‐month‐old mice. In panel F, ^a^ *P* < 0.01 vs CON at 6 months of intervention and ^b^ *P* < 0.01 and *P* < 0.0 5 vs CRL and CRS, respectively, in 18‐month‐old animals. In panel F, # denotes a positive linear trend (*P* < 0.001) of decreasing Nv in calorie‐restricted animals for 18 months (CRL > CRS > CRF).

The examination of thin sections of renal cortex from 6‐ and 18‐month‐old control and CR mice revealed among other structures a relatively high number of glomerular corpuscles as well as cross‐sectioned proximal convoluted tubules (PCT) displaying a typical structure with epithelial cells showing a well‐developed apical brush border, basal or central nuclei and a high number of mitochondria profiles. In Fig. S1 (Supporting information), we show one of these sections as an example of the materials used in this work. First, we performed a planimetric analysis of PCT epithelial cells and nuclei from the different diet groups. Epithelial cellular size decreased during aging in control mice but remained unaltered in the CRS group (Fig. 3A). However, when comparing cellular area among the different CR groups, CRF showed cell sizes significantly higher than those of CRL, which decreased during aging, and CRS at both 6 and 18 months (Fig. 3A). Neither aging nor diet affected nuclear size when comparing 6‐ versus 18‐month‐old mice in any diet group (Fig. 3B). Nevertheless, in 6‐month‐old animals some differences appeared when comparing the CR groups with CRS having the smallest and CRF the largest nuclei (Fig. 3B).

In PCT epithelial cells, mitochondria appear as numerous electron‐dense structures spread out throughout the cytoplasm regardless of mouse age or dietary group. Some examples of mitochondrial appearance in PCT cells are included in Fig. S2A–D (Supporting information). After planimetric and stereological analyses of mitochondria, striking differences were found in the experimental groups. Mean mitochondrial volume increased during aging in CON mice (Fig 3C), and the same effect was observed in the CRS group. CRL and CRF followed a nearly identical pattern to the CRS group. Similar changes were observed when comparing mitochondrial area and major and minor diameters from the 3 CR groups (not shown). Aging also induced a significant decrease in mitochondrial circularity coefficient in CON and CRS mice (Fig. 3D). In addition, significant differences were also found when comparing the CR groups of animals fed with the different dietary fats. In this case, circularity coefficient was higher in CRF compared to all the other CR groups (Fig 3D).

The stereological parameters volume density (Vv; i.e., cell volume fraction occupied by mitochondria) and numerical density (Nv; i.e., number of mitochondria per cell volume unit) also changed during aging and/or CR. In CON animals, aging resulted in a decrease of Vv, a phenomenon that was not observed in CRS mice (Fig. 3E). However, when comparing the different CR groups, a significant decrease of Vv was found in the CRL group while CRF remained unaltered compared to CRS (see Fig. 3E). Conversely, aging affected Nv in different ways depending on the experimental group. Thus, in CON mice Nv did not change significantly in 6‐ versus 18‐month‐old animals, but markedly decreased in CRS mice (Fig. 3F). In this group, we also found increased Nv after 6 months when compared to CON. When comparing Nv among the CR dietary groups at a given age, we found the highest values in CRS after 6 months of dietary intervention and in CRL after 18 months. In aged animals, a decreasing linear trend CRL > CRS > CRF was found for Nv (see Fig. 3F). Of note, statistical analyses showed a clear correlation between GBM thickness and mitochondrial Vv and Nv in PCT cells in such a way that a thicker GBM corresponded with lesser mitochondrial mass in PCT cells. A similar correlation was observed when comparing glomerular FS width and mitochondrial Vv in PCT cells (see Fig. S4D,E,F).

**Figure 4:**
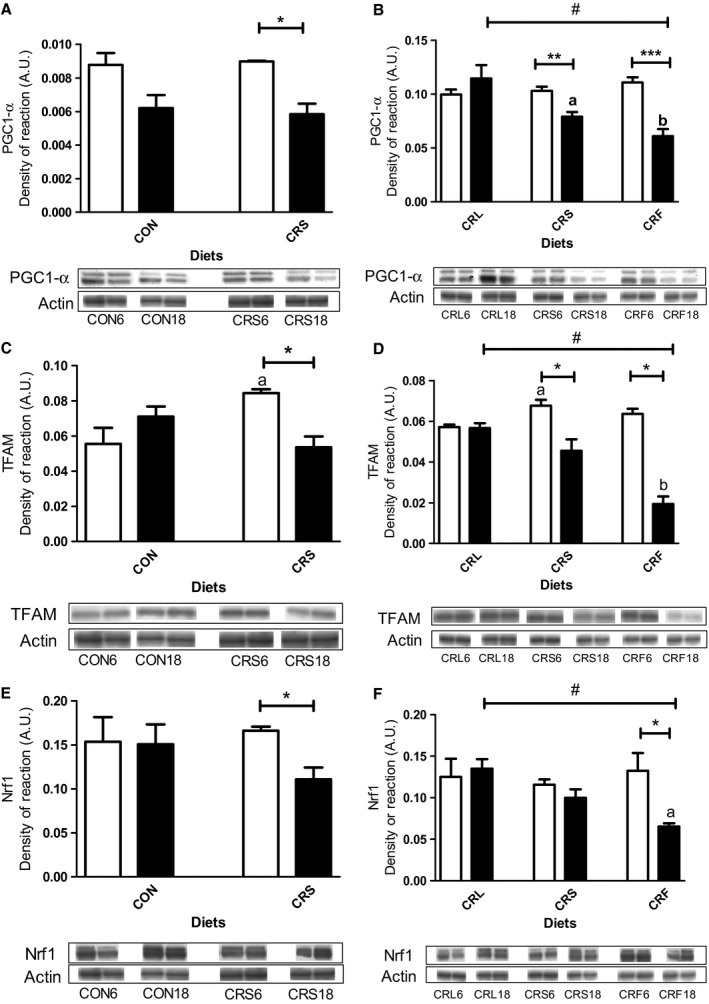
Representation of PGC1‐α (panels 4A and 4B), TFAM (panels 4C and 4D), and NRF1 (panels 4 E and 4F) protein expression levels in the different dietary groups during aging (*P < 0.05, **P < 0.01, and ***P < 0.001). In panel B, ^a^p < 0.05 vs CRL and ^b^ *P* < 0.01 vs CRL and *P* < 0.05 vs CRS in 18‐month‐old groups. In panel C, ^a^ *P* < 0.05 vs CON. In panel D, ^a^ *P* < 0.05 vs CRL after 6 months of CR and ^b^ *P* < 0.05 vs CRL and CRF in 18‐month CR mice. A decreasing linear trend (^#^ *P* < 0.01) was found in old CR animals CR (CRL > CRS > CRF) for PGC1‐α, TFAM, and NRF1 expression levels. Two representative Western blot bands for each experimental group are also shown.

The observation of mitochondrial size and mass variations among the different ages and diets led us to explore possible changes in mitochondrial dynamics and/or biogenesis, and thus, we performed an expression analysis of PGC‐1α, the regulatory master of these processes, and its downstream transcription factors NRF1 (nuclear respiratory factor 1) and TFAM (mitochondrial transcription factor A). Our results show that protein expression of all three proteins did not change significantly at 6 versus 18 months in control mice but were markedly decreased with aging in CRS mice (Fig 4A,C,E). After 6 months of CR, PGC‐1α, and NRF1 remained unaltered in all the three CR groups but TFAM increased significantly in CRS compared with CRL (Fig 4B,D,F). However, after 18 months of CR, the expression levels of PGC‐1α, TFAM and NRF1 were higher in CRL (*P* < 0.01) compared to CRS and CRF (see Fig 4B,D,F).

### Autophagy ultrastructural observations

**Figure 5:**
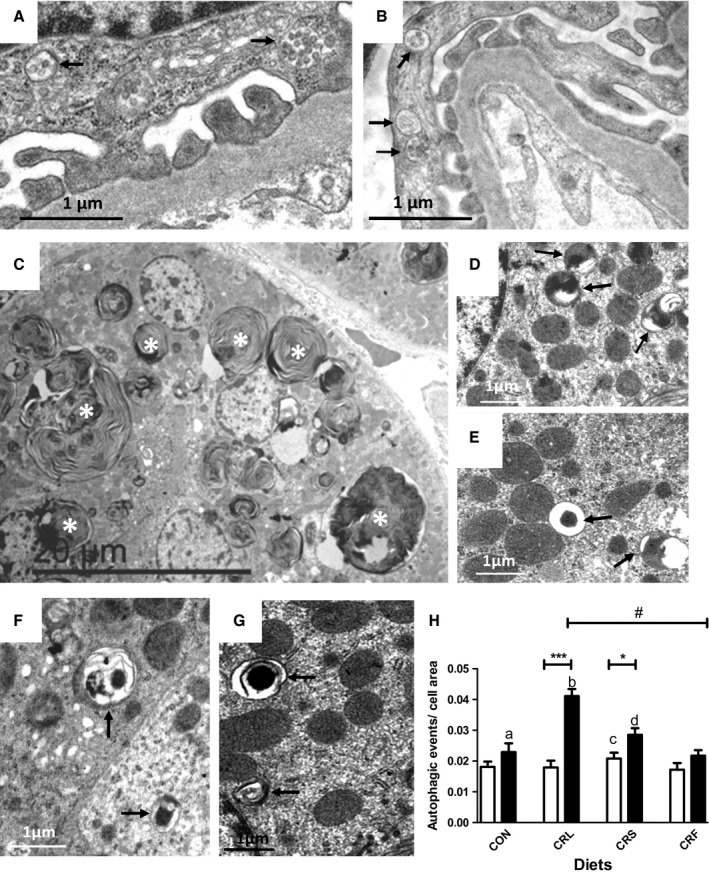
Ultrastructural localization of autophagic figures (arrows) in 18‐month control or CR mice (A, C, and D = CON; B and F = RCL; E = CRS; and G = RCF). Pictures A and B are podocytes from CON and CRL groups, respectively. Proximal convoluted tubular cells also showed autophagic figures regardless the dietary fat (Panels D, E, F, and G). In CON mice (panel C), a relatively high number of PCT showed an elevated number of enlarged lysosomes with characteristic concentric lamellar inclusions (asterisks). The results of a quantification of number of autophagic event figures in relation to cell area are shown in panel H (^a^ *P* < 0.05 vs CRS 18 months; ^b^ *P* < 0.01 vs CRS and CRF in 18‐month mice;^c^ *P* < 0.05 vs CRF in six‐month intervention; ^d^ *P* < 0.05 vs CRF 18 months). A positive linear trend of decreasing autophagic events in calorie‐restricted animals for 18 months (CRL > CRS > CRF) was also found (^#^ *P* < 0.001).

Electron microscopy images showing typical structures of autophagy (see, for example, Hartleben *et al*., 2010 and Kume *et al*., 2010) were found in podocytes (Fig. 5A,B) and in PCT epithelial cells (Fig. 5C–G). These figures consisted of cytoplasmic portions surrounded by membranes showing irregular shape and content which frequently appeared as typical myelinlike figures. Although these structures appeared in all of the experimental groups regardless of age or feeding condition, they were more abundant in 18‐month‐old animals (Fig. 5A–H). In some cases, PCT showed considerable sized myelinlike structures occupying most of the cellular space (see Fig 5C). A quantitative analysis of these figures at the electron microscopy level yielded the results displayed in Fig. 5H. After 6 months of dietary intervention, a similar number of autophagic events appeared in CON and CRS mice. However, after 18 months of CR, this parameter significantly increased in CRS in comparison with CON. When comparing the different CR groups, we found no changes during aging in CRF and a considerable increase in CRL, which was more prominent that in CRS (see Fig. 5H). After 18 months of CR, we found a statistically significant linear trend ordered as CRL > CRS > CRF (see Fig. 5H).

### Western blot analysis of autophagy markers

**Figure 6:**
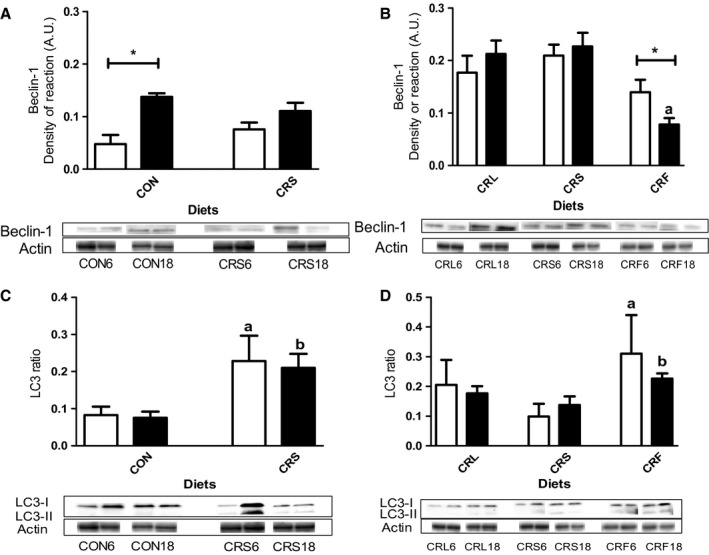
Representation of Beclin‐1 expression levels (Panels A and B) and LC3 ratio (LC3‐II/LC3‐II + LC3‐I; panels C and D) in the dietary groups. In panels A and C, we represent young and old controls and CR animals with soybean as dietary fat for Beclin‐1 (*P < 0.05) and LC3 ratio, respectively (^a,b^ *P* < 0.05 vs respective CON group). In panel B and D, we represent the effect of 6 and 18 months of CR with the different fat sources on proteins expression levels (Panel B: ^a^ *P* < 0.05 vs RCS and *P < 0.05; panel D: ^a,b^ *P* < 0.05 vs respective CRS animals). In all panels, two representative Western blot bands for each experimental group are shown.

The expression levels of two autophagy markers were investigated during aging and CR with different dietary fats: Beclin‐1 and LC3. The results obtained for Beclin‐1 expression are shown in Fig. 6A,B. Aging induced a significant increase of this marker in control animals, but no changes were observed in the CRS group (see Fig. 6A). When we analyzed the effects of long‐term CR with the different dietary fats, we found decreased expression levels of this protein in CRF mice (Fig. 6B). No age‐related changes were observed in the other CR groups. The lowest expression levels of Beclin‐1 corresponded to those found in CRF after 18 months of CR and were significantly decreased in comparison with CRS mice (Fig. 6B).

Changes in expression levels of LC3 were also evaluated. The pre‐LC3 form is cleaved into the cytosolic form LC3‐I, which is then conjugated to phosphatidylethanolamine to form LC3‐II (see, for example, Cui *et al*., 2012, 2013). The ratio LC3‐II/LC3‐I + LC3‐II is correlated with autophagic flux. LC3 ratio remained unchanged in CON or CRS animals during aging. However, a significant increase LC3 ratio was observed in the CRS compared to CON group (Fig. 6C). When comparing the effects of the dietary fats, we found similar levels of LC3 ratio at 6 and 18 months in the CRL and/or CRS groups. However, CRF animals showed increased ratios at 6 and 18 months of CR when compared to the corresponding CRS groups (Fig. 6D).

## Discussion

We have recently shown that 40% CR extends lifespan in mice. However, differences in longevity were found among CR groups depending on the source of dietary fat. Thus, lard extended longevity compared to soy and fish oils (López‐Domínguez *et al*., 2015a) pointing to a role of specific dietary components in determining lifespan of mice fed CR diets. To assess the precise effects of this nutritional intervention at a tissue level, we analyzed the impact of dietary fat in different organs and tissues from animals fed these same diets (see Khraiwesh *et al*., 2013 and Khraiwesh *et al*., 2014 and López‐Domínguez *et al*., 2013, 2015b). In this paper, we studied kidney structure and biology in an attempt to determine the possible role this organ plays in health and aging in CR mice fed diets that differ in fat composition.

As occurs in other tissues and organs of mammals, kidney undergoes physiological and morphological changes during aging that lead to its deterioration and a consequent decline in renal function (Martin & Sheaff, 2007; Bolignano *et al*., 2014). Although CR had an important effect of decreasing serum urea content, no differences were found due to dietary fat. Also, creatinine clearance was similar in all experimental groups. These results are in accordance with those reported in rats as no striking changes in both metabolites were detected in aged *ad libitum* or CR‐fed rats (Cui *et al*., 2012, 2013; Ning *et al*., 2013). On the other hand, we show here that p16 expression increased in CRS group during aging but maintained lesser values than those obtained for CON animals, which is in accordance with the results reported by other authors (Cui *et al*., 2012, 2013). Diet lipid composition did not impact age‐related changes in p16 expression in CR mice.

Morphological and biochemical changes in glomeruli and PCT have been described in aged kidney. These changes include increased glomerulosclerosis, podocyte loss, GBM thickening, accumulation of abnormal mitochondria, and alteration in autophagy (Lindeman & Goldman, 1986; McKiernan *et al*., 2007; Bolignano *et al*., 2014). Most of these changes have been observed in our samples in all of the dietary groups, but especially in the CON animals. However, differences were found in CR groups depending on the dietary fat.

Although the proportion of sclerotic glomeruli was similar in CON and CR mice after 18 months of intervention, we found pronounced changes in nonaltered glomeruli which affected the glomerular basement membrane (GBM) thickness, the separation between contiguous podocyte processes (filtration slits, FS) and podocyte foot processes (PFP) width in the zone of contact with the GBM. These structures are essential components of the renal filtration barrier. Strikingly, GBM thickness increased after 6 months of CR when soy or fish oil were the primary dietary fats in comparison with CRL animals. However, 18 months of CR resulted in a remarkable increase of this parameter in all of the experimental groups. Wiggins *et al*. (2005) found similar results in rats subjected to CR and suggested that the increase in GBM thickness is an age‐associated phenomenon and largely unrelated to diet. Although our results partially fit with these observations, it was noticeable that GBM thickness did not increase to the same extent in all of CR groups, with CRL‐fed mice showing lesser values compared to all other CR groups, indicating that under CR dietary fat may partially prevent the increase in GBM thickness during aging.

Filtration slits width decreased during aging in control mice, a phenomenon that was prevented in CRS‐fed animals. Moreover, our results show a negative correlation between GBM thickness and FS width in our animal model. On the other hand, it has been shown that aging induces expansion of podocyte processes (Wiggins *et al*., 2005; Hartleben *et al*., 2010), a phenomenon that could result in the reduction of FS width. In our control animals, aging also induced podocyte processes expansion, a phenomenon that was partially prevented by CR. However, dietary fat greatly affected this parameter. Thus, PFP width increased in aged CRF mice but remained unaltered in CRL‐fed animals. Furthermore, our results show a negative correlation between PFP and FS width indicating that the narrowed FS found during aging can be due to PFP expansion. We also found a positive correlation between PFP width and GBM thickness during aging, two phenomena considered as hallmarks of glomerular aging (Wiggins *et al*., 2005; McKiernan *et al*., 2007; Hartleben *et al*., 2010; Bolignano *et al*., 2014). To our best knowledge, this is the first report showing a narrowing in FS which correlated with increased GBM thickness and PFP expansion during aging. However, further studies will be necessary to elucidate the physiological significance of this relationship. Also, the effects of specific dietary fats on GBM, FS, and PFP sizes have not been previously reported, and the results of the present study point out a possible role of dietary fat in the maintenance of these structures under CR conditions.

Aging also affected mitochondrial morphology and mass in PCT cells from mice submitted to CR. With the exception of CRL‐fed mice, aging resulted in increased mitochondrial volume and decreased circularity. These results were especially prominent in 18‐month CRS and CRF mice in which mitochondrial volumes increased nearly 45% in comparison with their younger counterparts. These results are in line with those obtained for mitochondrial Nv and the expression levels of PGC‐1α, the key master of mitochondrial biogenesis regulation, and its downstream targets NRF1 and TFAM. Thus, low expression of these proteins was found in those groups showing enlarged mitochondria and low Nv values (CRS and CRF), and higher expression levels were detected in CRL group in which we found the smallest mitochondria and the highest Nv value. These results together with those concerning cellular and nuclear size, likely indicate differential adaptation mechanisms to the conditions imposed by CR. Interestingly, the response of PCT cells (in terms of cell and nuclear size and mitochondrial mass) to the different dietary fats under CR described here was similar to that found in mice hepatocytes (Khraiwesh *et al*., 2013, 2014).

In an attempt to link glomerular ultrastructural changes with changes in epithelial PCT cells, several analyses were performed. Thus, we found a negative correlation between GBM thickness and mitochondrial mass (Vv and Nv) in PCT cells in such a way that a thick GBM corresponded to less mitochondrial mass in PCT cells. A similar negative correlation was also found when comparing FS width and Vv in PCT cells. These results seem to point out an adaptive response of PCT cells to changes in renal glomerular structures imposed by aging.

One of the hallmarks of aging is the generation of damaged organelles and molecular aggregates which may be removed by autophagy, and the decline in autophagic capacity is involved in the development of age‐related diseases (see, for example, Rajawat *et al*., 2009). In kidney, decreased autophagy has been related to glomerulosclerosis, tubular atrophy, and interstitial fibrosis, and two different renal cell types have been reported to display autophagic activity depending on the physiological conditions: podocytes and PCT cells (Hartleben *et al*., 2010; Kume *et al*., 2010). In rodent kidney, aging has been shown to decrease phagocytic activity, a phenomenon that can be partially reverted by CR (Kume *et al*., 2010; Cui *et al*., 2012, 2013). In our model, Beclin‐1, a protein involved in the control of autophagosome formation, increased during aging in CON animals. Among CR mice, Beclin‐1 remained unaltered with age except in the CRF group in which a significant decrease was found.

The ratio of LC3‐II/LC3‐I has been shown to be an effective marker of autophagic flux. In our study, aging had no effect on this parameter in CON animals but markedly increased after 6 months of CR and these results are in accordance to those reported by several authors (Kume *et al*., 2010; Cui *et al*., 2012; Ning *et al*., 2013). Long‐term CR did not induce additional increase of LC3 ratio in CRS group, although this value remained higher than that found in old CON mice. When comparing the different dietary fats in CR mice, we found the highest LC3 ratios in CRF group for both 6 and 18 months of CR. These results seem to indicate differential regulatory mechanisms of autophagy during calorie restriction depending on the fat source.

At the electron microscopy level, we found typical figures of autophagy in podocytes and PCT cells, especially in old animals. However, enlarged structures consisting of multiple concentrically arranged electron‐dense lamella occupying a high proportion of cellular volume were mainly found in PCT cells from old control and CRF‐fed mice. Nearly identical structures have been reported in a C57BL/6 mouse model of accelerated renal senescence (Yumura *et al*., 2006) and in other murine models with defective lysosomal activity (Porubsky *et al*., 2014). These authors identify these structures as lysosomes with accumulated lipofuscin and perhaps other nondegradable pigments, an idea compatible with the well‐known fact that PCT cells may accumulate lipofuscin during aging (Melk *et al*., 2003). Furthermore, it has been proposed that a positive correlation exists between damaged mitochondria, lipofuscin accumulation and aging (Brunk & Terman, 2002). Due to its role in solute reabsorption, PCT cells show a high number of mitochondria making feasible the presence of these structures in this cell type as a consequence of altered autophagy or mitophagy processes for mitochondrial renewal. As PCT cells show a high rate of turnover (Fougeray & Pallet, 2015), it is not possible to assess whether proximal tubules showing an elevated number of altered lysosomes are fated to loss or regeneration.

As dietary lipids (lard, soybean oil, and fish oil) used in this and in previous studies are complex, comparisons between dietary lipids can be hampered by the fact that the lipids differ in multiple fatty acids. Thus, it cannot be unequivocally concluded which specific fatty acids were responsible for the biochemical and structural differences observed between CR groups. Nevertheless, we have previously shown that mitochondrial phospholipid fatty acid composition was altered in liver and skeletal muscle from CR mice in a manner that reflected the unsaturated fatty acid composition of the diet with the consequent increase of n‐3 and n‐6 fatty acids in CRF‐ and CRS‐fed animals, respectively, and probably changing several properties of the membranes (Chen *et al*., 2012, 2013). On the other hand, CRL‐fed animals showed a significantly higher proportion of mitochondrial monounsaturated fatty acids (especially oleic acid), a result that was accompanied by improved mitochondrial functions and ultrastructure (see Villalba *et al*., 2015 for a recent review). Thus, it is very likely that an increase in monounsaturated fatty acids such as oleic acid may be involved in the beneficial effect of lard as a dietary fat in CR‐fed animals. However, further studies with purified fatty acids will be required to identify the specific fatty acids which influence health and lifespan in CR mice.

In summary, in this paper we report that long‐term CR partially prevents or delays the appearance of several structural hallmarks of aging kidney, such as enlargement of GBM and PFP, FS narrowing, or PCT cells modification. However, these effects differed depending on the dietary fat. CRL mice showed an improved preservation of several renal structures (GBM thickness, PFP width, mitochondrial mass, size and shape, and autophagic processes in PCT cells) compared to other diet groups. These results fit well with those reported by López‐Domínguez *et al*. (2015a) in which CR using lard as fat source resulted in extended longevity in comparison with other dietary fat (soy and fish oil), reinforcing the idea that dietary fat may have a crucial role in the determination of CR‐mediated healthy aging in mice.

## Experimental procedures

### Animals and diets

A cohort of 64 ten‐week‐old male C57BL/6 mice was used (Charles River Laboratories, Wilmington, MA, USA). Mice were bred and raised in a *vivarium* at the *Centro Andaluz de Biología del Desarrollo* (CABD, Sevilla, Spain) under a 12‐h light/dark cycle (8:00 a.m.–8:00 p.m.) and at controlled conditions of temperature (22 ± 3 °C) and humidity. The mice were fed a commercial rodent chow diet (Harlan Teklad #7012, Madison, WI, USA) for 14 days, and then, the animals were randomly assigned into four dietary groups and were fed a modified AIN‐93G purified diet. The control group was fed 95% of a predetermined *ad libitum* intake (12.5 kcal). This slight restriction in food intake was initiated to prevent excessive weight gain during the study. The three CR dietary groups were maintained on 60% of the daily allowance of the control intake (8.6 kcal), and these diets were identical except for dietary lipid sources. The diets (percent total kilocalories per day) contained 20.3% protein, 63.9% carbohydrate, and 15.8% fat. The dietary fat for the control group was soybean oil. Dietary fats for the three CR groups were soybean oil (high in n‐6 PUFAs, Super Store Industries, Lathrop, CA, USA), fish oil (high in n‐3 PUFAs: 18% EPA, 12% DHA, Jedwards International, Inc. Quincy, MA, USA), or lard (high in saturated and monounsaturated fatty acids, ConAgra Foods, Omaha, NE, USA). To insure adequate linoleic acid levels, the CR‐fish group was supplemented with soybean oil. Fatty acid composition of the dietary lipids has been detailed in a separate publication (Chen *et al*., 2012). All mice were housed individually and were fed with control or CR diets for 6 or 18 months, respectively. Filtered and acidified water was available *ad libitum* for all groups, and food was replaced every day between 8:00 and 9:00 a.m.

At either 6 or 18 months of CR, the animals were weighed and sacrificed by cervical dislocation after fasting O/N (or 12 h). Kidneys were quickly dissected and processed for ultrastructural analysis, and homogenates were also prepared for protein expression studies. Blood was collected by cardiac puncture just after cervical dislocation. Serum was obtained by centrifugation in Vacuette Z serum Sep Clot activator tubes for 10 min at 3000 *g* and stored in small aliquots kept at −80 °C until the determination of different blood metabolites. Serum creatinine was determined using an ELISA KIT (Alpha diagnostic international, San Antonio, Texas, USA) as indicated by the manufacturer. Urea was determined using the Reflotron plus system (Roche, Basel, Switzerland). All experimental procedures and animal handling were in accordance with the Pablo de Olavide University Ethical Committee rules, and the 86/609/EEC Directive on the protection of animals used for experimental and other scientific purposes.

### Electron microscopy, planimetric and stereological analysis

Small pieces from renal cortex were fixed and embedded in epoxy resin by conventional methods (see Data S1). The blocks were sectioned to obtain semi‐thick (0.5–1 μm thickness) and thin (40–60 nm) sections. In semi‐thick sections, we analyzed renal glomeruli morphology and thin sections were viewed and photographed for other measurements. GBM thickness, filtration slits (FS), and podocyte foot processes (PFP) width were measured using the imagej software (N.I.H.; Bethesda, MD, USA). Planimetric mitochondrial measurements of PCT cells were performed on pictures containing whole cells (see Figs S1 and S2) and using ImageJ software. From the same pictures, we obtained the mitochondrial stereological parameters Volume density (Vv) and Numerical density (Nv) using the semi‐automatic application ‘WimStereology’ (Wimasis SL, Córdoba, Spain), based on a simple square lattice test system (Weibel, 1979). The relative number of autophagosomes and autophagic‐related figures per cell surface area was also scored for each dietary group. Detailed information on ultrastructural procedures and applications is included in the Data S1 (Supporting information).

### Tissue processing for Western blotting analysis

Kidneys were homogenized following a common protocol (see Data S1) to obtain properly samples for Western blot analysis. The samples were loaded in SDS‐PAGE and then transferred into nitrocellulose sheets. The quantification of the load was measured with Ponceau S to carry out the normalization of the film bands (see Fig. S3). On the sheets, we performed an immunostaining for p16, PGC 1‐α, NRF1, TFAM, Beclin‐1, and LC3 I/II using appropriated primary and secondary antibodies that were revealed with horseradish peroxidase on a photographic film. Detailed information is included in the Data S1 (Supporting information).

### Statistical analysis

Values were expressed as mean ± SEM. D'Agostino–Pearson tests were performed to determine data normality. The effect of CR was assessed by Student's *t*‐test (CRS group vs Control). In case the data did not pass the normality test, the nonparametric Mann–Whitney test was followed. The effect of dietary fat under CR was assessed by one‐way ANOVA followed by a *post hoc* analysis (Tukey's test for multiple comparisons) to assess significant differences among groups. *Post hoc* analysis of linear trend was also performed to investigate putative alterations of tested parameters among CR diets ordered as CRL→CRS→CRF, which resulted in a progressive increase of the n‐6/n‐3 ratio in phospholipid highly unsaturated fatty acids (Chen *et al*., 2012). In case the data did not pass the normality test, the nonparametric Kruskal–Wallis test was followed. Correlation analyses were performed by the nonparametric Spearman test. Means were considered statistically different at *P* < 0.05. All statistical analyses were performed using graphpad prism 5.03 (GraphPad Software Inc., San Diego, CA, USA).

## Authors contributions

JJR, PN, RdC, and JMV designed and supervised research; PN and GL‐LL maintained the mice colony; MC‐R, GL‐LL, MIB, JMV, and JAG‐R performed the experiments and analyzed the data; and JAG‐R wrote the manuscript.

## Funding

Supported by NIH grant 1R01AG028125 (to JJR, PN, and JMV), Ministerio de Economía y Competitividad BFU2011‐23578 (to JMV) and DEP2012‐39985 (to GL‐L), Junta de Andalucía Proyectos de Excelencia grant P09‐CVI‐4887 (to JMV), Junta de Andalucía Proyectos Internacionales (to JMV), BIO‐276 (Junta de Andalucía and the University of Córdoba, to JMV) and Fondo de Investigaciones Sanitarias FIS PI14‐01962 (to PN). RdC is supported by the Intramural Research Program of the National Institute on Aging of the National Institutes of Health. MC‐R is funded by predoctoral fellowship of the Spanish Ministerio de Educación and by BIO‐276.

## Conflict of interest

None declared.

## Supporting information


**Data S1** Supplementary methods.

**Fig. S1** Cross‐section of a proximal convoluted tubule (PCT) from a six‐month old control animal.

**Fig. S2** Representative images of cytoplasm portions of PCT epithelial cells from control (A) and 18‐months CR‐submitted animals with different dietary fats (B, CRL; C, CRS and D, CRF) showing a relatively large number of mitochondria (arrows). In C and D swollen mitochondria are clearly visible. The bars are equal to 2 µm (N = nucleus).

**Fig. S3** Representative gels stained with Ponceau S used to normalize quantifications of the different antibody bands shown in this paper.

**Fig. S4** Correlation analyses between different glomerular filtration structures (panels A, B and C) and glomerular structures *versus* mitochondrial mass in epithelial cells from proximal convoluted tubules (D, E and F). Panel A shows filtration slits (FS) *versus* glomerular basal membrane (GBM) thickness; panel B, podocyte foot processes (PFP) *versus* GBM and panel C, PFP *versus* FS. Panel D depicts GBM thickness *versus* mitochondrial volume density (Vv) in PCT cells; panel E, GBM thickness *versus* mitochondrial numerical density in PCT cells and panel E, PFP width *versus* mitochondrial Vv in PCT cells. In panel A, *P* < 0.001; in panels B‐E, *P* < 0.05. In this figure C is CON and L, S and F are CRL, CRS and CRF respectively. Number 6 and 18 indicates the duration of dietary intervention period.

## References

[acel12451-bib-0001] Bolignano D , Mattace‐Raso F , Sijbrands EJ , Zoccali C (2014) The aging kidney revisited: a systematic review. Ageing Res. Rev. 14, 65–80. 24548926 10.1016/j.arr.2014.02.003

[acel12451-bib-0002] Brunk UT , Terman A (2002) The mitochondrial‐lysosomal axis theory of aging: accumulation of damaged mitochondria as a result of imperfect autophagocytosis. Eur. J. Biochem. 269, 1996–2002. 11985575 10.1046/j.1432-1033.2002.02869.x

[acel12451-bib-0003] Campisi J (2013) Aging, cellular senescence, and cancer. Annu. Rev. Physiol. 75, 685–705. 23140366 10.1146/annurev-physiol-030212-183653 PMC4166529

[acel12451-bib-0004] Chen Y , Hagopian K , McDonald RB , Bibus D , López‐Lluch G , Villalba JM , Navas P , Ramsey JJ (2012) The influence of dietary lipid composition on skeletal muscle mitochondria from mice following 1 month of calorie restriction. J. Gerontol. A Biol. Sci. Med. Sci. 67, 1121–1131. 22503990 10.1093/gerona/gls113 PMC3636677

[acel12451-bib-0005] Chen Y , Hagopian K , Bibus D , Villalba JM , López‐Lluch G , Navas P , Kim K , McDonald RB , Ramsey JJ (2013) The influence of dietary lipid composition on liver mitochondria from mice following 1 month of calorie restriction. Biosci. Rep. 33, 83–95. 23098316 10.1042/BSR20120060 PMC3522480

[acel12451-bib-0006] Chung KW , Kim DH , Park MH , Choi YJ , Kim ND , Lee J , Yu BP , Chung HY (2013) Recent advances in calorie restriction research on aging. Exp. Gerontol. 48, 1049–1053. 23201549 10.1016/j.exger.2012.11.007

[acel12451-bib-0007] Colman RJ , Anderson RM , Johnson SC , Kastman EK , Kosmatka KJ , Beasley TM , Allison DB , Cruzen C , Simmons HA , Kemnitz JW , Weindruch R (2009) Caloric restriction delays disease onset and mortality in rhesus monkeys. Science 325, 201–204. 19590001 10.1126/science.1173635 PMC2812811

[acel12451-bib-0008] Cuervo AM (2004) Autophagy: many paths to the same end. Mol. Cell. Biochem. 263, 55–72. 27520665 10.1023/B:MCBI.0000041848.57020.57

[acel12451-bib-0009] Cui J , Bai XY , Shi S , Cui S , Hong Q , Cai G , Chen X (2012) Age‐related changes in the function of autophagy in rat kidneys. Age (Dordr). 34, 329–339. 21455601 10.1007/s11357-011-9237-1 PMC3312632

[acel12451-bib-0010] Cui J , Shi S , Sun X , Cai G , Cui S , Hong Q , Chen X , Bai XY (2013) Mitochondrial autophagy involving renal injury and aging is modulated by caloric intake in aged rat kidneys. PLoS One 8, e69720. doi: 10.1371/journal.pone.0069720. 23894530 10.1371/journal.pone.0069720 PMC3718786

[acel12451-bib-0011] Fougeray S , Pallet N (2015) Mechanisms and biological functions of autophagy in diseased and ageing kidneys. Nat. Rev. Nephrol. 11, 34–45. 25385287 10.1038/nrneph.2014.201

[acel12451-bib-0012] González‐Freire M , de Cabo R , Bernier M , Sollott SJ , Fabbri E , Navas P , Ferrucci L (2015) Reconsidering the role of mitochondria in aging. J. Gerontol. A Biol. Sci. Med. Sci. 70, 1334–1342. 25995290 10.1093/gerona/glv070 PMC4612387

[acel12451-bib-0013] Hartleben B , Gödel M , Meyer‐Schwesinger C , Liu S , Ulrich T , Köbler S , Wiech T , Grahammer F , Arnold SJ , Lindenmeyer MT , Cohen CD , Pavenstädt H , Kerjaschki D , Mizushima N , Shaw AS , Walz G , Huber TB (2010) Autophagy influences glomerular disease susceptibility and maintains podocyte homeostasis in aging mice. J. Clin. Invest. 120, 1084–1096. 20200449 10.1172/JCI39492 PMC2846040

[acel12451-bib-0014] Hediger MA (2002) Kidney function: gateway to a long life? Nature 417, 393–395. 12024201 10.1038/417393a

[acel12451-bib-0015] Huber TB , Edelstein CL , Hartleben B , Inoki K , Jiang M , Koya D , Kume S , Lieberthal W , Pallet N , Quiroga A , Ravichandran K , Susztak K , Yoshida S , Dong Z (2012) Emerging role of autophagy in kidney function, diseases and aging. Autophagy 8, 1009–1031. 22692002 10.4161/auto.19821 PMC3429540

[acel12451-bib-0016] Hulbert AJ (2003) Life, death and membrane bilayers. J. Exp. Biol. 206, 2303–2311. 12796449 10.1242/jeb.00399

[acel12451-bib-0017] Jové M , Naudí A , Ramírez‐Núñez O , Portero‐Otín M , Selman C , Withers DJ , Pamplona R (2014) Caloric restriction reveals a metabolomic and lipidomic signature in liver of male mice. Aging Cell 13, 828–837. 25052291 10.1111/acel.12241 PMC4331741

[acel12451-bib-0018] Khraiwesh H , López‐Domínguez JA , López‐Lluch G , Navas P , de Cabo R , Ramsey JJ , Villalba JM , González‐Reyes JA (2013) Alterations of ultrastructural and fission/fusion markers in hepatocyte mitochondria from mice following calorie restriction with different dietary fats. J. Gerontol. A Biol. Sci. Med. Sci. 68, 1023–1034. 23403066 10.1093/gerona/glt006 PMC3738026

[acel12451-bib-0019] Khraiwesh H , López‐Domínguez JA , Fernández del Río L , Gutiérrez‐Casado E , López‐Lluch G , Navas P , de Cabo R , Ramsey JJ , Burón MI , Villalba JM , González‐Reyes JA (2014) Mitochondrial ultrastructure and markers of dynamics in hepatocytes from aged, calorie restricted mice fed with different dietary fats. Exp. Gerontol. 56, 77–88. 24704714 10.1016/j.exger.2014.03.023 PMC4104696

[acel12451-bib-0020] Kume S , Uzu T , Horiike K , Chin‐Kanasaki M , Isshiki K , Araki S , Sugimoto T , Haneda M , Kashiwagi A , Koya D (2010) Calorie restriction enhances cell adaptation to hypoxia through Sirt1‐dependent mitochondrial autophagy in mouse aged kidney. J. Clin. Invest. 120, 1043–1055. 20335657 10.1172/JCI41376 PMC2846062

[acel12451-bib-0021] Lindeman RD , Goldman R (1986) Anatomic and physiologic age changes in the kidney. Exp. Gerontol. 21, 379–406. 3545873 10.1016/0531-5565(86)90044-6

[acel12451-bib-0022] López‐Domínguez JA , Khraiwesh H , González‐Reyes JA , López‐Lluch G , Navas P , Ramsey JJ , de Cabo R , Burón MI , Villalba JM (2013) Dietary fat modifies mitochondrial and plasma membrane apoptotic signaling in skeletal muscle of calorie‐restricted mice. Age (Dordr). 35, 2027–2044. 23179253 10.1007/s11357-012-9492-9 PMC3824980

[acel12451-bib-0023] López‐Domínguez JA , Ramsey JJ , Tran D , Imai DM , Koehne A , Laing ST , Griffey SM , Kim K , Taylor SL , Hagopian K , Villalba JM , López‐Lluch G , Navas P , McDonald RB (2015a) The influence of dietary fat source on life span in calorie restricted mice. J. Gerontol. A Biol. Sci. Med. Sci. 70, 1181–1188. 25313149 10.1093/gerona/glu177 PMC4612357

[acel12451-bib-0024] López‐Domínguez JA , Khraiwesh H , González‐Reyes JA , López‐Lluch G , Navas P , Ramsey JJ , de Cabo R , Burón MI , Villalba JM (2015b) Dietary fat and aging modulate apoptotic signaling in liver of calorie‐restricted mice. J. Gerontol. A Biol. Sci. Med. Sci. 70, 399–409. 24691092 10.1093/gerona/glu045 PMC4375413

[acel12451-bib-0025] López‐Lluch G , Irusta PM , Navas P , de Cabo R (2008) Mitochondrial biogenesis and healthy aging. Exp. Gerontol. 43, 813–819. 18662766 10.1016/j.exger.2008.06.014 PMC2562606

[acel12451-bib-0026] López‐Otín C , Blasco MA , Partridge L , Serrano M , Kroemer G (2013) The hallmarks of aging. Cell 153, 1194–1217. 23746838 10.1016/j.cell.2013.05.039 PMC3836174

[acel12451-bib-0027] Madeo F , Zimmermann A , Maiuri MC , Kroemer G (2015) Essential role for autophagy in lifespan extension. J. Clin. Invest. 125, 85–93. 25654554 10.1172/JCI73946 PMC4382258

[acel12451-bib-0028] Martin J , Sheaff M (2007) Renal ageing. J. Pathol. 211, 198–205. 17200944 10.1002/path.2111

[acel12451-bib-0029] Mattison JA , Roth GS , Beasley TM , Tilmont EM , Handy AM , Herbert RL , Longo DL , Allison DB , Young JE , Bryant M , Barnard D , Ward WF , Qi W , Ingram DK , de Cabo R (2012) Impact of caloric restriction on health and survival in rhesus monkeys from the NIA study. Nature 489, 318–321. 22932268 10.1038/nature11432 PMC3832985

[acel12451-bib-0030] McKiernan SH , Tuen VC , Baldwin K , Wanagat J , Djamali A , Aiken JM (2007) Adult‐onset calorie restriction delays the accumulation of mitochondrial enzyme abnormalities in aging rat kidney tubular epithelial cells. Am. J. Physiol. Renal. Physiol. 292, F1751–F1760. 17344189 10.1152/ajprenal.00307.2006

[acel12451-bib-0031] Melk A , Kittikowit W , Sandhu I , Halloran KM , Grimm P , Schmidt BM , Halloran PF (2003) Cell senescence in rat kidneys in vivo increases with growth and age despite lack of telomere shortening. Kidney Int. 63, 2134–2143. 12753300 10.1046/j.1523-1755.2003.00032.x

[acel12451-bib-0032] Miquel J , Economos AC , Fleming J , Johnson JE Jr (1980) Mitochondrial role in cell aging. Exp. Gerontol. 15, 575–591. 7009178 10.1016/0531-5565(80)90010-8

[acel12451-bib-0033] Ning YC , Cai GY , Zhuo L , Gao JJ , Dong D , Cui S , Feng Z , Shi SZ , Bai XY , Sun XF , Chen XM (2013) Short‐term calorie restriction protects against renal senescence of aged rats by increasing autophagic activity and reducing oxidative damage. Mech. Ageing Dev. 134, 570–579. 24291536 10.1016/j.mad.2013.11.006

[acel12451-bib-0034] Pamplona R , Barja G , Portero‐Otín M (2002) Membrane fatty acid unsaturation, protection against oxidative stress, and maximum life span: a homeoviscous‐longevity adaptation? Ann. N. Y. Acad. Sci. 959, 475–490. 11976221 10.1111/j.1749-6632.2002.tb02118.x

[acel12451-bib-0035] Porubsky S , Jennemann R , Lehmann L , Gröne HJ (2014) Depletion of globosides and isoglobosides fully reverts the morphologic phenotype of Fabry disease. Cell Tissue Res. 358, 217–227. 24992926 10.1007/s00441-014-1922-9 PMC4186980

[acel12451-bib-0036] Rajawat YS , Hilioti Z , Bossis I (2009) Aging: central role for autophagy and the lysosomal degradative system. Ageing Res. Rev. 8, 199–213. 19427410 10.1016/j.arr.2009.05.001

[acel12451-bib-0037] Speakman JR , Mitchell SE (2011) Caloric restriction. Mol. Aspects Med. 32, 159–221. 21840335 10.1016/j.mam.2011.07.001

[acel12451-bib-0038] Villalba JM , López‐Domínguez JA , Chen Y , Khraiwesh H , González‐Reyes JA , Del Río LF , Gutiérrez‐Casado E , Del Río M , Calvo‐Rubio M , Ariza J , de Cabo R , López‐Lluch G , Navas P , Hagopian K , Burón MI , Ramsey JJ (2015) The influence of dietary fat source on liver and skeletal muscle mitochondrial modifications and lifespan changes in calorie‐restricted mice. Biogerontology 16, 655–670. 25860863 10.1007/s10522-015-9572-1 PMC4555094

[acel12451-bib-0039] Weibel ER (1979) Stereological Methods. Practical Methods for Biological Morphometry, vol. 1 New York, NY: Academic Press.

[acel12451-bib-0040] Weindruch RH , Walford RL (1988) The Retardation of Aging and Disease by Dietary Restriction. Springfield, IL: Charles C. Thomas.

[acel12451-bib-0041] Wiggins JE (2012) Aging in the glomerulus. J. Gerontol. A Biol. Sci. Med. Sci. 67, 1358–1364. 22843670 10.1093/gerona/gls157 PMC3636672

[acel12451-bib-0042] Wiggins JE , Goyal M , Sanden SK , Wharram BL , Shedden KA , Misek DE , Kuick RD , Wiggins RC (2005) Podocyte hypertrophy, “adaptation”, and “decompensation” associated with glomerular enlargement and glomerulosclerosis in the aging rat: prevention by calorie restriction. J. Am. Soc. Nephrol. 16, 2953–2966. 16120818 10.1681/ASN.2005050488

[acel12451-bib-0043] Youngman LD , Park JY , Ames BN (1992) Protein oxidation associated with aging is reduced by dietary restriction of protein or calories. Proc. Natl Acad. Sci. U.S.A. 89, 9112–9116. 1409611 10.1073/pnas.89.19.9112 PMC50075

[acel12451-bib-0044] Yu BP , Lim BO , Sugano M (2002) Dietary restriction downregulates free radical and lipid peroxide production: plausible mechanism for elongation of life span. J. Nutr. Sci. Vitaminol. (Tokyo) 48, 257–264. 12489815 10.3177/jnsv.48.257

[acel12451-bib-0045] Yumura W , Imasawa T , Suganuma S , Ishigami A , Handa S , Kubo S , Joh K , Maruyama N (2006) Accelerated tubular cell senescence in SMP30 knockout mice. Histol. Histopathol. 21, 1151–1156. 16874657 10.14670/HH-21.1151

