# Supplementary material for: Dietary fat composition influences glomerular and proximal convoluted tubule cell structure and autophagic processes in kidneys from calorie‐restricted mice
Source: Aging Cell. 2016 Feb 8;15(3):477–87. doi: 10.1111/acel.12451 (PMC4854917; doi:10.1111/acel.12451)
Supplement: Supplementary file 1 — Data S1 Supplementary methods. [file ACEL-15-477-s001.docx]

Supplementary methods

**Tissue processing for light and electron microscopy**

Kidneys were removed and quickly washed in 0.1 M sodium cacodylate buffer pH 7.2. Then they were cut in small pieces of about 1 mm^3^. For each animal, we processed up to seven pieces taken from the cortex area of the organ. Five to seven animals were processed in every diet and age group. Samples were fixed in a mixture of 2.5% glutaraldehyde-2% paraformaldehyde in sodium cacodylate buffer (0.1M; pH 7.2) for 6-8 hour and then washed in buffer and postfixed in 1% osmium tetroxide for 1 hour at 4^o^C in the same buffer. After dehydration in an ascendant series of ethanol, the pieces were transferred to propylene oxide and sequentially infiltrated in EMbed 812 resin (EMS; USA). We used the sequence propylene oxide-resin 2:1; 1:1, and 1:2 throughout 24 hour. Afterwards samples were transferred to pure resin for 24 hour. Blocks were formed in fresh resin contained in silicon molds, and the resin was then allowed to polymerize for 48 hour at 65 ^o^C. After trimming, blocks were sectioned in an Ultracut Reicher ultramicrotome to obtain both semi-thick (0.5-1 µm width) and ultrathin (40-60 nm width) sections.

**Glomerular study**

Semi-thick sections from at least two blocks of each animal were mounted on glass slides and stained with 1% toluidine blue in a 1% borax aqueous solution for 2 min. Micrographs were obtained using a Leica DME light microscope. About 50 glomeruli per dietary group were scored. Using the same blocks we also obtained ultrathin sections that were mounted on nickel grids. After staining in aqueous 4% uranyl acetate and lead citrate, the sections were observed and photographed in a Jeol Jem 1400 Transmission Electron Microscope. High magnification pictures of glomerular basement membrane (GBM) originally at 30,000X, were used to obtain the average thickness of the GBM as well as the filtration slit (FS) and podocyte foot processes (PFP) width. To get these parameters we obtained 8-10 pictures per glomeruli from about 3-4 glomeruli per resin block (6-8 animals per experimental group; two blocks per animal) showing unaltered morphology. Glomeruli displaying sclerotic symptoms were discarded. GBM thickness was measured every 0.5 µm along and perpendicularly to the GBM (GBM was considered as the space between the external plasma membrane of a capillary vessel and the perpendicular-fronted plasma membrane of a podocyte process; see Fig. 2E), and all of the visible FS and PFP were scored in the same pictures. We considered PFP width as the portion of PFP plasma membrane in contact with the GBM. Using this method we obtained measures of about 500 GBM and 450 FS and PFP per experimental group. Measures of GBM, FS and PFP were performed using ImageJ software (N.I.H.).

**Mitochondrial ultrastructure and stereology in proximal convoluted tubule (PCT) epithelial cells.**

From the same sections used above we obtained low-magnification micrographs (originally at 7,500X) containing whole epithelial cells from PCT in order to determine cellular and nuclear sizes. We analyzed about 40 cells for each dietary group. Cells were selected on the basis of showing central/basal well-developed nuclei. Cellular and mitochondrial planimetric parameters (area, major and minor diameters and circularity coefficient) and number of mitochondria per cell section were also obtained from these pictures. Mitochondrial volumes were calculated using the formula${V=\frac{4}{3} \pi\left( D/2 \right)(d/2)}^{2}$, being “D” and “d” major and minor diameter, respectively and assimilating mitochondrial shape to prolate spheroids. For planimetric measurements about 1,500 mitochondria per group were scored.

A stereological analysis was carried out in order to get information on mitochondrial volumetric parameters. The stereological parameters referred to mitochondria volume density (Vv) and numerical density (Nv) values were obtained throughout the automatic application “Wim Stereology” (Wimasis SL, Spain), based in the simple square-lattice test system (Weibel, 1979). Numerical density was calculated using the formula

$$Nv = \frac{k}{\beta}\frac{{Na}^{3/2}}{{Vv}^{1/2}}$$

where “Na” represents the number of mitochondria per μm^2^ of cell and “k” and “β” the mitochondrial size distribution and shape coefficient, respectively. These coefficients were calculated using the results of planimetric measurements (Weibel, 1979). Planimetric measurements on mitochondria were performed using the same automatic software.

Finally, using the same pictures of PCT cells mentioned above, we scored the relative number of autophagosomes and autophagic-related figures per cell surface area.

**Kidney homogenization**

Kidneys were minced and homogenized in ice-cold isolation buffer containing 5 mMTris-HCl pH 7.4, 0.225 M mannitol, 0.075 M sucrose, 0.5 mM ethylene glycol tetraacetic acid, 1 mM phenylmethylsulfonyl fluoride, and 20µg/µl each chymostatin, leupeptin, antipain, and pepstatin with the aid of a Teflon-glass tissue homogenizer. After that, an aliquot from the total homogenate was re-homogenized using a mechanical tissue disrupter (Ultra-Turrax T25, IKA; Staufen, Germany) during 30s and then stored at -80 ^o^C until further analysis.

**Western Blotting analysis**

About 50 µg of protein was denatured by heating in SDS-dithiothreitol loading buffer [10% sucrose, 2m MEDTA, 1.5% (w/v) SDS, 20 mM dithiothreitol, 0.01% (w/v) bromophenol blue, and 60 mM Tris-HCl, pH 6.8], separated by SDS-PAGE (12.5% acrylamide) and then blotted onto nitrocellulose sheets. Blots were stained with Ponceau S for visualization of protein lanes. P16, PGC1-α, LC3 I/II and Beclin-1 polypeptides were measured in whole homogenates. Polypeptide detection was carried out by immunostaining of Western blots respectively with a mouse anti-p16 antiserum (Santa Cruz Biotechnology, Inc) diluted at 1:1,000, a rabbit anti-PGC1-α antiserum (Santa Cruz Biotechnology, Inc) diluted at 1:2,000, a rabbit anti-NRF1 antiserum (Santa Cruz Biotechnology, Inc) diluted at 1:1,000, a goat anti-TFAM antiserum (Santa Cruz Biotechnology, Inc) diluted at 1:1,000, a rabbit anti-LC3 I/II antiserum (Cell Signaling, Inc) diluted at 1:1000, a goat anti-Beclin-1 antiserum (Santa Cruz Biotechnology, Inc) diluted at 1:1,000 and a rabbit anti-actin antiserum (Sigma-Aldrich) diluted at 1:1,000. The corresponding secondary IgG antibodies coupled to horseradish peroxidase (Sigma) were used to reveal binding sites by enhanced chemiluminiscence (ECL-Plus, GE Healthcare Life Sciences).

Photographic films and Ponceau S-stained blots were scanned in a GS-800 calibrated densitometer (Bio-Rad) to obtain digital images. Quantification of intensity reaction was carried out using Quantity One software (Bio-Rad). Data obtained from the quantification of the stained bands (in arbitrary units) were normalized to those of the corresponding lane stained with Ponceau S in order to correct any difference in protein loading between samples. In order to have an accurate estimation of changes produced by CR per se and by alterations of dietary fat in CR animals, the effects of these two dietary manipulations were assessed in separate electrophoresis gels and blots carried out under optimized conditions for each case. Thus, results of protein levels measured by Western blotting were also represented in separate plots: one for CR effect (CON vs CRS) and the other one for dietary fat effects in CR animals (CRL, CRS and CRF; see Supplementary Figure 3).
