# Supplementary figures and images for: Dietary fat composition influences glomerular and proximal convoluted tubule cell structure and autophagic processes in kidneys from calorie‐restricted mice
Source: Aging Cell. 2016 Feb 8;15(3):477–87. doi: 10.1111/acel.12451 (PMC4854917; doi:10.1111/acel.12451)

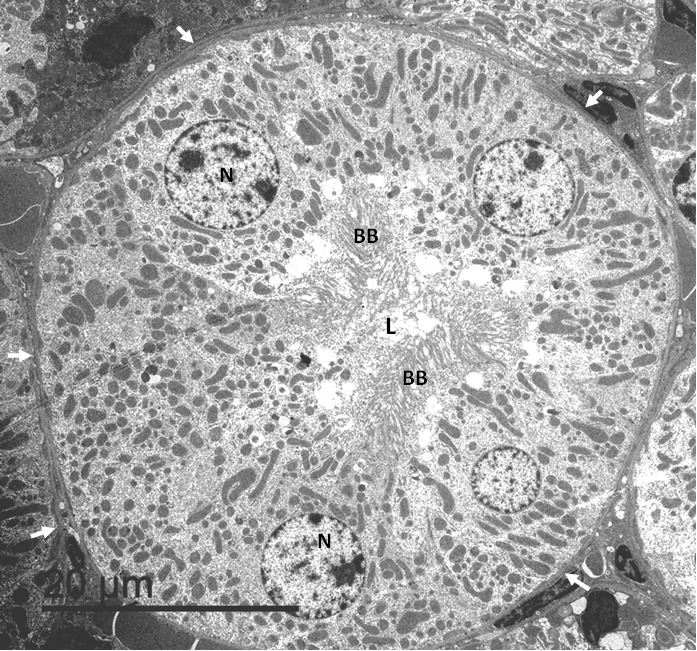

Supplement: Supplementary file 2 — Fig. S1 Cross‐section of a proximal convoluted tubule (PCT) from a six‐month old control animal. [file ACEL-15-477-s002.tif]

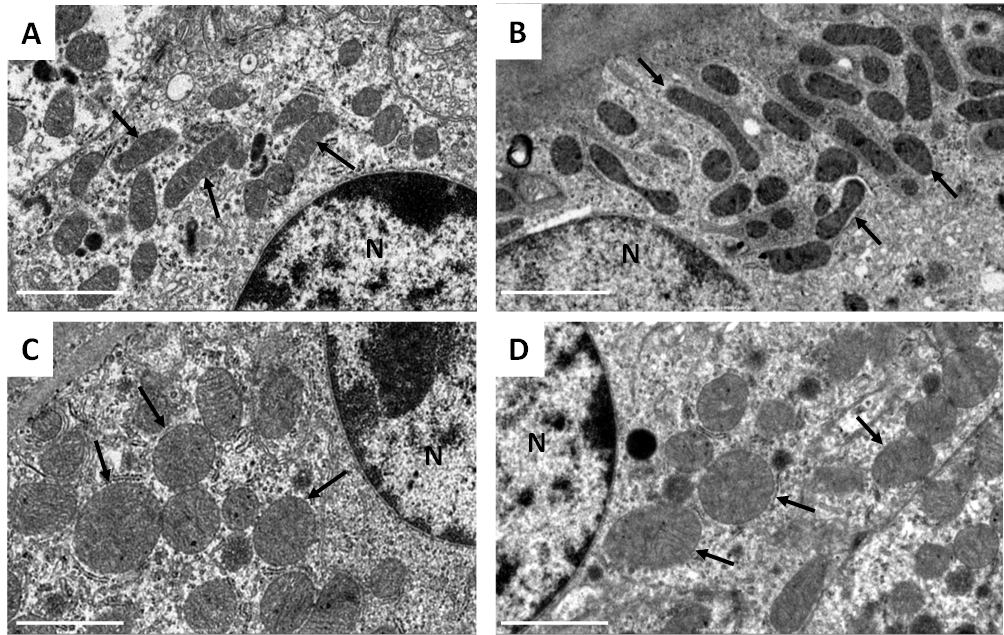

Supplement: Supplementary file 3 — Fig. S2 Representative images of cytoplasm portions of PCT epithelial cells from control (A) and 18‐months CR‐submitted animals with different dietary fats (B, CRL; C, CRS and D, CRF) showing a relatively large number of mitochondria (arrows). In C and D swollen mitochondria are clearly visible. The bars are equal to 2 µm (N = nucleus). [file ACEL-15-477-s003.tif]

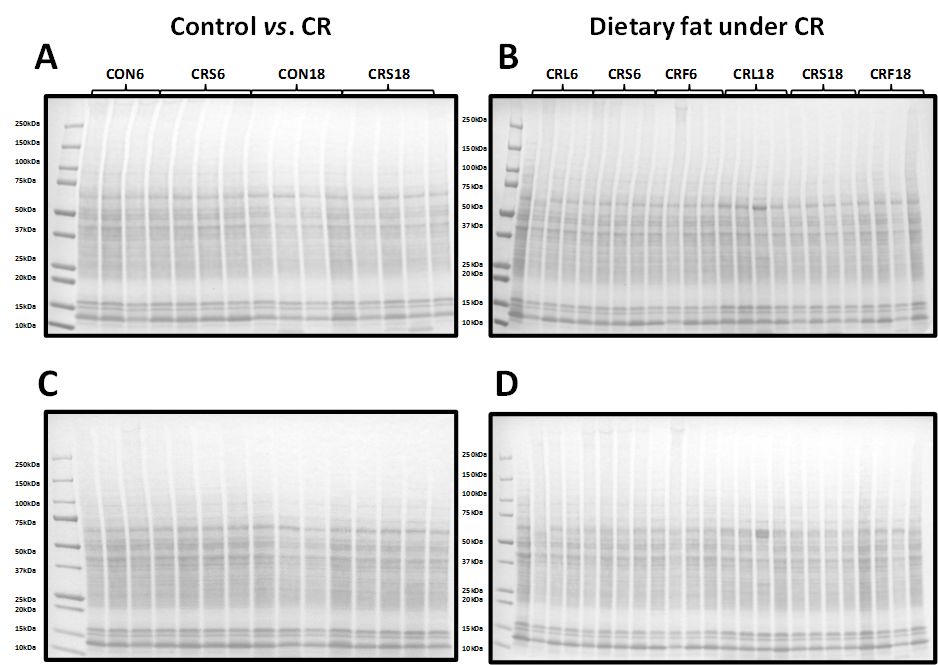

Supplement: Supplementary file 4 — Fig. S3 Representative gels stained with Ponceau S used to normalize quantifications of the different antibody bands shown in this paper. [file ACEL-15-477-s004.tif]

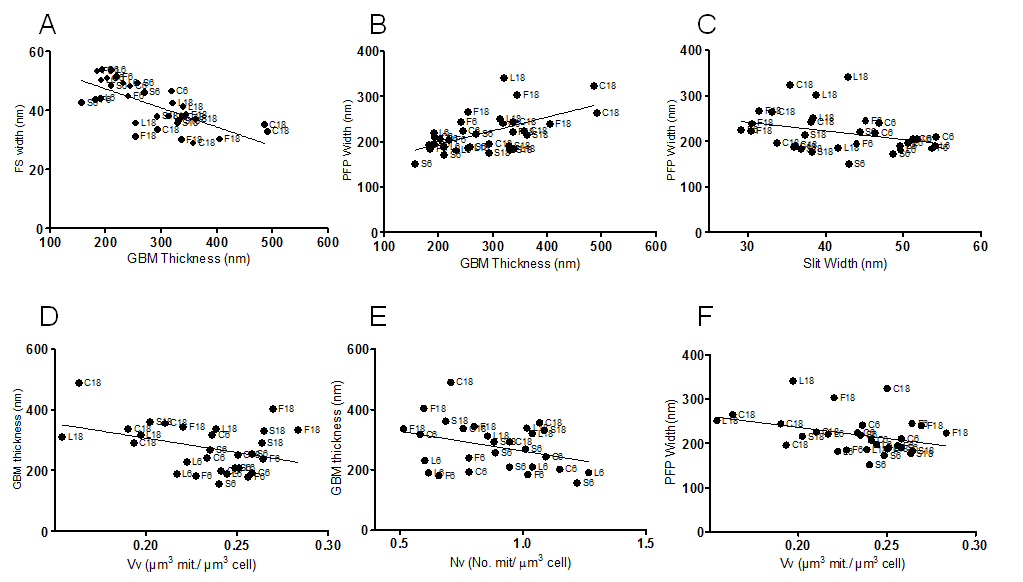

Supplement: Supplementary file 5 — Fig. S4 Correlation analyses between different glomerular filtration structures (panels A, B and C) and glomerular structures versus mitochondrial mass in epithelial cells from proximal convoluted tubules (D, E and F). Panel A shows filtration slits (FS) versus glomerular basal membrane (GBM) thickness; panel B, podocyte foot processes (PFP) versus GBM and panel C, PFP versus FS. Panel D depicts GBM thickness versus mitochondrial volume density (Vv) in PCT cells; panel E, GBM thickness versus mitochondrial numerical density in PCT cells and panel E, PFP width versus mitochondrial Vv in PCT cells. In panel A, P < 0.001; in panels B‐E, P < 0.05. In this figure C is CON and L, S and F are CRL, CRS and CRF respectively. Number 6 and 18 indicates the duration of dietary intervention period. [file ACEL-15-477-s005.tif]
