# Supplementary material for: Dietary fat composition influences glomerular and proximal convoluted tubule cell structure and autophagic processes in kidneys from calorie‐restricted mice
Source: Aging Cell. 2016 Feb 8;15(3):477–87. doi: 10.1111/acel.12451 (PMC4854917; doi:10.1111/acel.12451)
Supplement: Supplementary file 6 [file ACEL-15-477-s006.docx]

Supplementary Figure legends

Supplementary Figure 1.- Cross-section of a proximal convoluted tubule (PCT) from a six-month old control animal. A basal lamina (arrows) limits the epithelial cell at the basal border. The apical domain of epithelial cells is characterized by a well-developed brush border (BB) with a high number of microvilli partially occluding the tubular lumen (L). Epithelial cells show central or basal positioned nuclei (N) and a high number of spherical and/or elongated mitochondria.

Supplementary Figure 2.- Representative images of cytoplasm portions of PCT epithelial cells from control (A) and 18-months CR-submitted animals with different dietary fats (B, CRL; C, CRS and D, CRF) showing a relatively large number of mitochondria (arrows). In C and D swollen mitochondria are clearly visible. The bars are equal to 2 µm (N= nucleus).

Supplementary Figure 3.- Representative gels stained with Ponceau S used to normalize quantifications of the different antibody bands shown in this paper. Panels A and C show control *versus* calorie restricted mice; panels B and D represent the different dietary fats under calorie restriction. Panels A and B were used to normalize anti-P16, anti-PGC-1α and anti-Beclin1 and panels C and D were used to normalize Nrf1, TFAM and LC3-I/LC3-II.

Supplementary Figure 4.- Correlation analyses between different glomerular filtration structures (panels A, B and C) and glomerular structures *versus* mitochondrial mass in epithelial cells from proximal convoluted tubules (D, E and F). Panel A shows filtration slits (FS) *versus* glomerular basal membrane (GBM) thickness; panel B, podocyte foot processes (PFP) *versus* GBM and panel C, PFP *versus* FS. Panel D depicts GBM thickness *versus* mitochondrial volume density (Vv) in PCT cells; panel E, GBM thickness *versus* mitochondrial numerical density in PCT cells and panel E, PFP width *versus* mitochondrial Vv in PCT cells. In panel A, p < 0.001; in panels B-E, p < 0.05. In this figure C is CON and L, S and F are CRL, CRS and CRF respectively. Number 6 and 18 indicates the duration of dietary intervention period.
